# Professional practice model for a hospital network: Nursing methodology research 

**DOI:** 10.15649/cuidarte.4348

**Published:** 2025-04-24

**Authors:** Doris Helena Torres – Acosta, Sandra Patricia Pulido – Barragán, Edwin Darío Archila – Hernández, Olga Lucía Laverde – Contreras, Beatriz Sánchez – Herrera

**Affiliations:** 1 Hospital Universitario de La Samaritana, Bogotá, Colombia. enfermeria.subdirect@hus.org.co Hospital Universitario de La Samaritana Hospital Universitario de La Samaritana Bogotá Colombia enfermeria.subdirect@hus.org.co; 2 Hospital Universitario de La Samaritana, Bogotá, Colombia. enfermeria.liderdp@hus.org.co Hospital Universitario de La Samaritana Hospital Universitario de La Samaritana Bogotá Colombia enfermeria.liderdp@hus.org.co; 3 Hospital Universitario de La Samaritana, Bogotá, Colombia. Estudiante de Doctorado en Enfermería Universidad de La Sabana. edwinarhe@unisabana.edu.co Hospital Universitario de La Samaritana. Hospital Universitario de La Samaritana. Bogotá Colombia edwinarhe@unisabana.edu.co; 4 Universidad de La Sabana. Chía-Cundinamarca Colombia. lulagreen28@gmail.com Universidad de La Sabana Universidad de La Sabana Chía-Cundinamarca Colombia lulagreen28@gmail.com; 5 Universidad de La Sabana. Chía-Cundinamarca Colombia. publifer@unisabana.edu.co Universidad de La Sabana Universidad de La Sabana Chía-Cundinamarca Colombia publifer@unisabana.edu.co

**Keywords:** Nursing Methodology Research, Leadership, Nursing Theory, Education, Nursing, Patient Safety, Patient-Centered Care, Compassion, Investigación Metodológica en Enfermería, Liderazgo, Teoría de Enfermería, Educación en Enfermería, Seguridad del Paciente, Atención Dirigida al Paciente, Compasión, Pesquisa Metodológica em Enfermagem, Liderança, Teoria de Enfermagem, Educação em Enfermagem, Segurança do Paciente, Assistência Centrada no Paciente, Compaixão

## Abstract

**Introduction:**

The emergence of hospital networks requires nursing models to guide practice and research.

**Objective:**

To develop a professional practice model to guide nursing care practice, teaching, and research in a teaching hospital network.

**Materials and Methods:**

This study involved Nursing Methodology Research conducted within an academic-service partnership over three years in a hospital network in Colombia. It followed a humanistic theoretical framework and a collective construction technique with guideline-based development and analysis. It included four consecutive phases: 1) Identification of the need for the model within the Hospital Network, 2) recognition of nursing metaparadigm concepts for practice, 3) formulation and prioritization of the assumptions required to achieve nursing mission and vision, and 4) consolidation, sharing, and validation of the model. A total of 156 out of the 185 nurses in the organization participated.

**Results:**

The model developed for the Hospital Network consists of three essential components: compassion, safe care, and nursing leadership.

**Discussion:**

The collective participation of the nursing staff and the work made within the academic-service partnership facilitated the adoption of the model. This development responds to the Hospital Network's strategic guidelines for quality of care, includes internal and external validation, and adheres to international standards.

**Conclusion:**

The "Leadership in Compassionate and Safe Care" nursing practice model guides, facilitates, and makes visible teaching and care practice and nursing research inside and outside of the Hospital Network.

## Resumen

**Introducción:**

El surgimiento de redes hospitalarias requiere tener modelos de enfermería que orienten su práctica e investigación.

**Objetivo:**

Desarrollar un modelo de práctica profesional para orientar la asistencia, la docencia y la investigación de enfermería en una red hospitalaria de carácter universitario.

**Materiales y métodos:**

Esta es una Investigación metodológica en enfermería desarrollada en alianza docencia servicio del 2021-2023, en una Red Hospitalaria en Colombia. Acogió un marco teórico humanístico y siguió una técnica de construcción colectiva con desarrollo y análisis basado en guías. Incluyó cuatro fases consecutivas: 1. Identificación de la necesidad del modelo para la Red 2. Reconocimiento de conceptos meta paradigmáticos de enfermería en la práctica. 3. Planteamiento y priorización de los supuestos requeridos para poder lograr la misión y visión de enfermería; y 4. Consolidación, socialización y validación del modelo. Participaron 156 de los 185 enfermeros de la organización.

**Resultados:**

El modelo desarrollado para la Red consta de tres componentes esenciales: la compasión, el cuidado seguro y el liderazgo de enfermería.

**Discusión:**

La participación colectiva del personal de enfermería y el trabajo en alianza docencia – servicio facilitaron la apropiación del modelo. Este desarrollo responde a lineamientos estratégicos de calidad del cuidado de la Red, incluyó validación interna y externa y cumple estándares internacionales.

**Conclusiones:**

El modelo de práctica de enfermería Liderazgo en cuidado compasivo y seguro orienta, facilita y permite hacer visible la práctica docente y asistencial y la investigación de enfermería en y desde la Red.

## Resumo

**Introdução:**

O surgimento de redes hospitalares exige modelos de enfermagem que orientem sua prática e pesquisa.

**Objetivo:**

Desenvolver um modelo de prática profissional para orientar a assistência, o ensino e a pesquisa de enfermagem em uma rede hospitalar universitária.

**Materiais e métodos:**

Trata-se de uma pesquisa metodológica em enfermagem desenvolvida em regime de parceria ensino-serviço no período de 2021 a 2023, em uma Rede Hospitalar da Colômbia. Adotou um referencial teórico humanístico e seguiu uma técnica de construção coletiva com desenvolvimento e análise baseados em guias. Contemplou quatro fases consecutivas: 1. Identificação da necessidade do modelo para a Rede 2. Reconhecimento dos conceitos metaparadigmáticos da enfermagem na prática. 3. Declaração e priorização dos pressupostos necessários para atingir a missão e a visão da enfermagem; e 4. Consolidação, socialização e validação do modelo. Participaram 156 dos 185 enfermeiros da organização.

**Resultados:**

O modelo desenvolvido para a Rede consiste em três componentes essenciais: compaixão, cuidado seguro e liderança de enfermagem.

**Discussão:**

A participação coletiva da equipe de enfermagem e o trabalho em aliança entre ensino e serviço facilitaram a apropriação do modelo. Este desenvolvimento responde às diretrizes estratégicas da Rede para a qualidade do atendimento, incluindo validação interna e externa, e está em conformidade com os padrões internacionais.

**Conclusões:**

O modelo de prática de enfermagem Liderança em Cuidados Compassivos e Seguros orienta, facilita e possibilita a visibilidade do ensino e da prática assistencial e da pesquisa em enfermagem na e a partir da Rede.

## Introduction

According to the World Health Organization, quality of care refers to the extent to which health services can achieve desired outcomes^1^. Nursing's commitment to quality healthcare has been widely documented^2^. To ensure the best possible quality service, nursing has proposed several strategies that include, but are not limited to: strengthening nursing theoretical models^3^,^4^; strengthening methodologies^5^,^6^ and professional competencies^7^; designing indicators with technological support to monitor practice outcomes^8^; and strengthening leadership and work teams^9^,^10^. 

Within the nursing theoretical models, professional practice models have been emphasized as important to focus and ensure quality care across different levels of care. These models respond to the metaparadigm or universal agreement that nursing should focus on caring for individuals' health experiences wherever they are^11^. Professional practice models are associated with professional development and nursing leadership at the institutional level^12^. These models have proven valuable in implementing and improving nursing practice within networked health systems^13^. They have also been a valuable guide for nursing education and research^14^. 

The recently consolidated teaching Hospital Network located in Colombia, where the study was conducted, required the establishment of unified guidelines for practice, teaching, and research. These guidelines aimed to ensure an optimal experience of both users and nursing staff, improve clinical outcomes, and optimize service costs. Therefore, the need arose to develop a professional nursing practice model that respond to the Hospital Network's specific characteristics and needs. 

## Materials and Methods

This study involved Nursing Methodological Research conducted within an academic-service partnership between 2021 and 2023. It sought to develop a professional nursing practice model for a newly created hospital network to guide nursing practice and teaching, as well as research^15^,^16^. The Hospital Network comprises two tertiary care hospitals (high complexity), one secondary care hospital (medium complexity), and two primary care health centers (low-complexity). 

The research observed the humanistic theoretical framework that guides the Hospital Network's services. It employed a collective construction technique, with development and analysis based on five guidelines. The model's preliminary proposal was validated by a group of experts and analyzed it against international standards for this type of theoretical development. Its phases were developed sequentially, as follows: 

**First**, identification of the need for a model for the Hospital Network. A research group was formed consisting of nine nurses, seven nursing leaders of the Hospital Network, and two university professors working in the field of nursing professional practice models. Following Guideline 1: “identification of the need for a professional practice model,” the group reviewed relevant institutional information, including the mission and vision of the newly established Hospital Network. Based on this, the desired future for nursing in the Hospital Network was discussed and shared with the nursing staff of each institution, and an action plan was jointly developed to achieve it. 

**Second**, using Guideline 2, “Recognition of nursing metaparadigm concepts for practice,” these concepts were identified. To achieve this, the coordinating group invited and included all the professional nurses within the Hospital Network, 185 in total, to respond individually. Their answers were recorded in an Excel workbook for subsequent qualitative analysis of the content and extraction of its essence^17^. 

**Third**, using two guidelines: Guideline 3, “Formulation of the assumptions required to fulfill the nursing mission and achieve the nursing vision,” and Guideline 4, “Prioritization of assumptions for the achievement of the nursing mission and vision.” In this phase, in order to ensure the broadest possible coverage, nine groups of registered nurses were formed, representing different services within the Hospital Network. Based on their input, the research group extracted the main assumptions of nursing care for analysis, removing those repeated from the final list. Subsequently, the groups were asked to prioritize the assumptions from the refined list based on their criteria by rating them quantitatively according to their level of importance and governance for nursing on a scale of 0 to 500 each. For the quantitative analysis, the research group compiled responses in an Excel spreadsheet to calculate the numerical averages of each assumption weighted between the two ratings given. They were then grouped into high, medium, or low priority levels according to their rating analysis and possible impact on achieving the vision and the institutional development plan. 

**Fourth**, the research group completed Guideline 5, “Consolidation, validation, and sharing of the model,” and used it as a basis for extracting the essence of the institution's professional practice model. To communicate the model following the steps of the guideline, the research group named it, developed a slogan and created an illustrative representation. 

The initial model version was validated internally by nursing staff across the Hospital Network to verify whether the model reflected their perceptions and priorities regarding nursing work in the Hospital Network. The adjusted version underwent external validation with the University’s nursing care study group, which verified its content. Lastly, the model’s degree of alignment with international standards for this type of theoretical nursing development, as summarized by Im^18^, was analyzed. 

Finally, the model was presented to hospital management and other units of the Hospital Network. The project's ethical approval act is 003 dated 14 03 2019. All collected data are freely accessible for consultation on Mendeley Data^19^. 

## Results

**Phase 1.** The common scenario to achieve the mission and vision of the new Hospital Network must reflect its humanistic nature. This requires a nursing staff whose attention is focused on service users, including patients and their families, and human resources in training. Nursing staff should show leadership, uphold ethical, respectful, and supportive behavior, be capable of teamwork, and reflect a vocation of service. Nursing practice must be based on the best available evidence and committed to quality in terms of safety, timeliness, continuous improvement, and the appropriate use of technology in each case. To achieve this purpose, a nursing model to guide nursing performance within the Hospital Network was deemed indispensable. 

**Phase 2.** The content analysis of the responses, which 87.00% of the Hospital Network's nursing staff provided, reflected four essential nursing concepts for the Hospital Network: subject of care, goal of nursing or health, context of care, and way of looking at nursing work (see Table 1). 

**Table 1:** Nursing metaparadigm concepts identified for the Hospital Network

Metaparadigm concept	Vision of each concept in the Hospital Network
Subject of care	For nurses within the Hospital Network, the subject of care is regarded as an individual or collective being and recognized as a patient, family caregiver, family, or community. The subject may be at different life cycle stages and receiving care from different services for different reasons. Often, they are vulnerable and require care. Members of the healthcare team are also regarded as subjects of care.
Health	For nurses within the Hospital Network, health is the well-being condition of the subjects of care. It requires satisfaction with the care and strengthening their ability to take care of their own health. Achieving the well-being of subjects of care means improving their health condition or quality of life, strengthening their autonomy, and promoting their prompt and full reintegration into society. Achieving this goal of care allows nurses to grow personally and professionally.
Context of Care	Nursing care is delivered within the context of a teaching hospital network that offers various basic and specialized healthcare services. This Hospital Network seeks to have a comprehensive bio-psycho-social and spiritual approach, individually or collectively focusing on people within the framework of a pleasant environment. It promotes quality care and knowledge development amid diversity. Altogether, the Hospital Network seeks to maintain health, strengthen recovery, alleviate suffering, and reduce the burden of disease for patients, their families, and caregivers.
Nursing	The role of nurses within the Hospital Network is to provide care for people by strengthening a caring bond with them. Recognition of others, a cordial approach, an inclusive attitude, and continuous supportive behavior characterize nurses' actions. Nurses promote a wholeness view and help to strengthen the autonomy of those who interact in caring. Nurses exercise their leadership role to fulfill their own or collaborative activities and coordinate management that promotes safe care based on the best available evidence.

**Phase 3.** From the groups' input, which included 51 nurses representing their respective services, the research group identified 52 assumptions of nursing care for the Hospital Network. These assumptions were then rated according to their importance and governance for nursing. Based on this input, the research group compared them with the results from Phase 1 and determined that, to fulfill the mission and vision of nursing within the Hospital Network, 16 assumptions were of high priority, 19 were of medium priority, and 17 were of low priority (see Table 2). 

**Figure 1:**
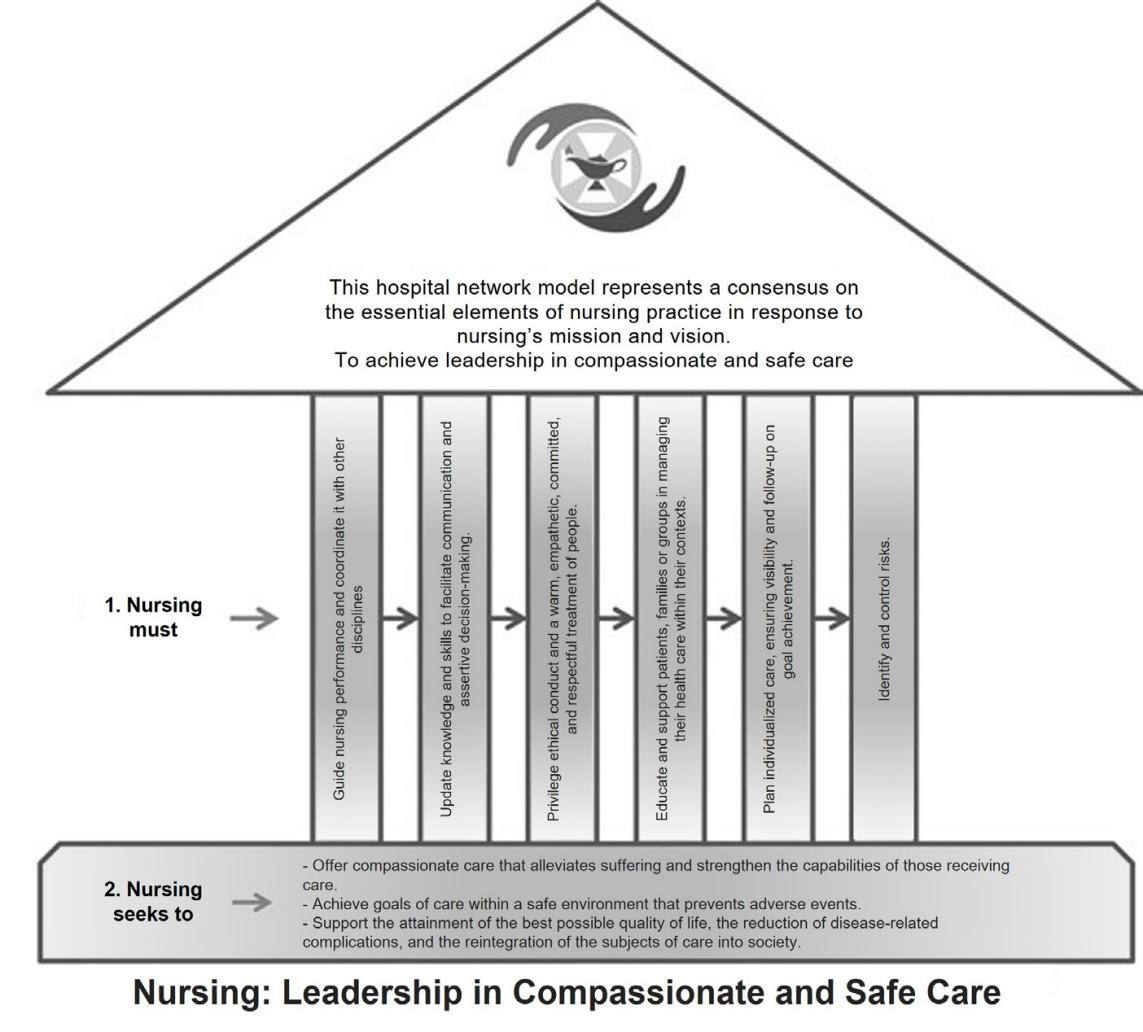
Diagram of the Leadership in Compassionate and Safe Care model

**Figure 2:**
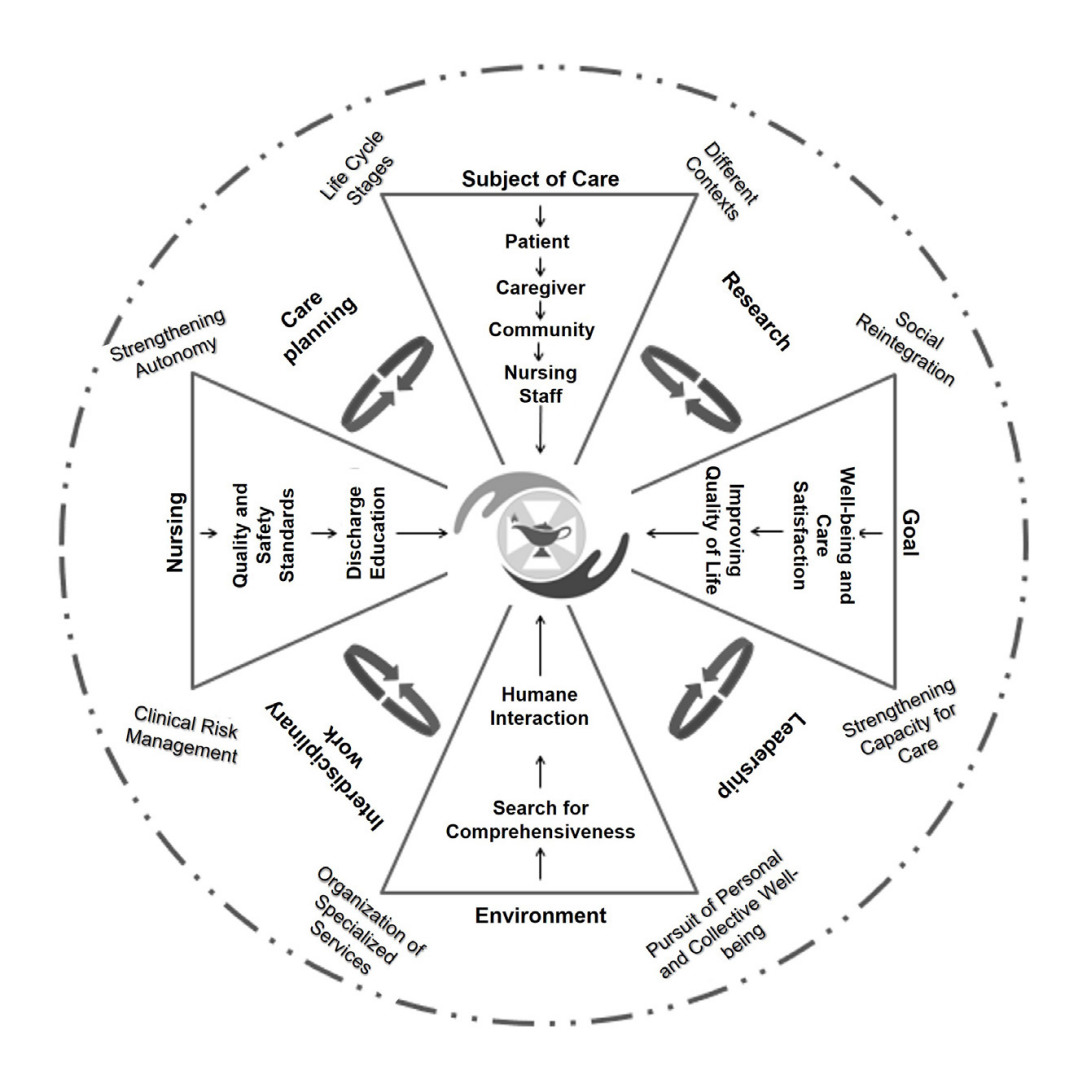
Visual representation of the components of the Leadership in Compassionate and Safe Care model

**Phase 4.** The professional nursing practice model was named “Leadership in Compassionate and Safe Care.” As its name suggests, this model aims to guide nursing practice within the Hospital Network to offer compassionate care, characterized by a cordial approach, an inclusive attitude, and supportive behavior. This care approach alleviates suffering and strengthens the capabilities of those receiving care or education while nurses strengthen themselves as caregivers. To achieve this, nurses must foster a safe environment that prevents adverse events. It is through care that nurses within the Hospital Network exercise leadership with an integrative approach that helps individuals being cared for promote health, prevent disease, facilitate healing, reduce complications, fully reintegrate into society, or achieve the best possible quality of life. These principles were visually represented by the coordinating group to graphically summarize the model’s approaches (see Figure 1 and 2). 

**Table 2:** Assumptions of care identified and prioritized for the Hospital Network

Priority level for mission and vision of nursing within the Hospital Network /Assumption	No.	IMP*	GV**	x̅	SD***
High priority					
The suitable environment for patient and family care within the Hospital Network requires nursing professionals who exercise leadership.	17	490	438	464.0	37.22
In the Hospital Network, good nursing care requires confidentiality criteria and adequate and timely records of the procedures performed on patients.	10	490	437	463.5	37.84
In the Hospital Network, nurses' cordial and safe approach must be part of their high standards of care.	26	481	440	460.5	28.53
To facilitate interdisciplinary work focused on the subject of care (patient, family, or community) within the Hospital Network, nurses must possess knowledge and leadership.	20	482	437	459.5	32.25
An adequate caring environment within the Hospital Network requires nurses’ commitment, ethics, knowledge, and skills.	23	484	435	459.5	34.74
Assertive communication and knowledge of nursing education are necessary for strengthening the capacity for care of patients and family members within the Hospital Network.	27	487	428	457.5	41.56
Within the Hospital Network, timely identification of risks affecting patients or their family caregivers allows for reducing and preventing adverse events.	51	482	432	457.0	35.36
Within the Hospital Network, quality nursing care requires nurses to have an empathetic and respectful attitude toward subjects of care, colleagues, and other institution members.	22	486	425	455,5	42,80
Within the Hospital Network, a caring environment requires nurses to focus on the subject of care (patient, family, or community) while maintaining a respectful relationship with colleagues and other professionals.	21	484	424	454.0	42.80
To achieve a safe environment within the Hospital Network, having assertive nursing professionals in decision-making is necessary.	18	480	425	452.5	38.46
The nursing care plan is the best tool nursing has within the Hospital Network for establishing, implementing, and meeting the goals of care.	43	472	433	452.5	27.29
Within the Hospital Network, humane care requires nursing to focus its practice on the subjects of care (patient, family, or community).	19	477	422	449.5	39.08
Nursing professionals in the Hospital Network should educate and raise awareness among patients and their families about the importance of self-care, enhancing quality of life, facilitating integration into society, and reducing disease-related complications.	48	481	418	449.5	44.66
For the subjects of care in the Hospital Network to improve their quality of life, nursing care must encourage, promote, and strengthen autonomy through support, education, treatment and rehabilitation support, respect, and cordiality.	49	475	419	447.0	39.08
Within the Hospital Network, nurses should allow space for patients and families to participate, ask questions, and express emotions that will facilitate ownership over and strengthen their healthcare.	1	474	419	446.5	38.46
To provide adequate nursing care in the Hospital Network, staff must be kind and knowledgeable about the different stages of the life cycle and the context.	7	482	407	444.5	53.34
In the Hospital Network, the well-being and satisfaction of patients, caregivers, family members, or community groups rely on ensuring humane nursing care that is technically and scientifically grounded.	50	477	411	444.0	47.14
Medium priority					
Within the Hospital Network, the environment should favor nursing leadership in supporting patient recovery.	14	475	412	443.5	44.04
Nursing care in the Hospital Network must uphold a scientific and moral commitment to preserving the best possible quality of life.	47	476	410	443.0	47.14
To ensure adequate nursing care in the Hospital Network, the subject of care (patient, user, family member, or community) must be viewed holistically using humanization criteria to strengthen their autonomy.	8	472	411	441.5	58.93
The Hospital Network's status as a teaching hospital should help nurses grow personally and professionally so that they can enhance the care they provide every day.	52	483	400	441.5	43.42
Within the Hospital Network, respect for privacy, beliefs, and culture, as well as a comprehensive approach with permanent strengthening of self-care, are essential elements for achieving the goals of care of those receiving assistance.	35	478	404	441.0	52.10
To provide good care to the subjects in the Hospital Network, ongoing training for nursing staff is required.	5	488	393	440.5	66.99
To strengthen the autonomy of the Hospital Network users, nursing must ensure their safety and well-being.	33	479	401	440.0	55.20
In the Hospital Network, nursing care focuses on providing self-care guidance to patients and their families to help them improve their lifestyles and promote health.	38	469	411	440.0	41.56
In the Hospital Network, nurses need to communicate assertively with the subject of care, establish direct contact to assess their condition, identify their needs, establish a plan that is jointly implemented, and evaluate it to verify the achievement of established goals regarding healthcare	41	471	403	437.0	55,82
Within the Hospital Network, a pleasant environment is required to promote the well-being of patients and their families during their hospital stay.	29	480	392	436.0	48.38
Nursing within the Hospital Network should focus care on strengthening people's autonomy as a contribution to improving their health condition, level of well-being, and quality of life.	30	474	398	436.0	62.03
To exercise nursing with leadership in clinical management within the Hospital Network, ongoing training is necessary.	13	482	389	435.5	53.34
Within the Hospital Network, nurses must model the way of caring so that nurses in training strengthen their capacity for care.	42	475	396	435.5	65.13
The palliative care required by patients and their families at the end of life demands nursing in the Hospital Network to establish specific goals of care that respond comprehensively to this particular situation.	37	476	392	434.0	56.44
Within the Hospital Network, the rational use of resources should be part of the responsibilities of nursing care.	46	468	400	434.0	59.55
Within the Hospital Network, the impact of nursing engagement through good care must extend beyond the hospital stay.	36	466	401	433.5	49.00
In the Hospital Network, the transcendent sense of care must be expressed through nursing interactions that can strengthen the subjects of care and positively impact their quality of life.	45	468	399	433.5	47.76
In the Hospital Network, organizational culture and adherence to manuals, procedures, technical procedures, instructions, and care protocols should enable quality care provision.	15	476	387	431.5	45.90
Low priority					
To provide adequate care in any service within the Hospital Network, nurses must seek to improve their quality of life and strengthen their capacity for care to support people's reintegration into society.	25	463	400	431.5	44.66
The pleasant environment of the Hospital Network should favor a holistic view and holistic care of human beings by nurses.	16	468	394	431.0	52.72
To permanently improve the quality of nursing care within the Hospital Network, it is necessary to establish and periodically evaluate specific indicators that measure nursing care.	24	469	386	427.5	58.93
Within the Hospital Network, the interdisciplinary healthcare team must be in charge of delivering comprehensive care and educating patients and families from admission through recovery, rehabilitation, and reintegration into the community.	40	468	387	427.5	57.69
Within the Hospital Network, as a university institution, nursing research should strengthen and innovate practices and methodologies to benefit the subject of care.	44	468	387	427.5	57.06
Within the Hospital Network, holistic care requires nursing personnel with a professional attitude, qualifications, strong ethical principles, adaptability, and ability to meet the needs of individuals or groups under care.	6	482	372	427.0	39.70
Within the Hospital Network, when nursing care successfully strengthens the autonomy and improves the quality of life of those receiving care, the professional discipline advances.	12	455	399	427.0	77.53
The Hospital Network must ensure nursing care in a safe environment that minimizes risks and promotes the well-being of individuals and groups.	4	475	378	426.5	68.23
To continuously improve the quality of nursing care in the Hospital Network, assessing individuals’ satisfaction with the care received is necessary.	11	472	368	420.0	73.81
Within the Hospital Network, by strengthening users’ capacity for care, nursing contributes to their quality of life and reintegration into society.	39	464	375	419.5	63.27
A comprehensive, culturally appropriate health education program that addresses Hospital Network users' needs helps to strengthen their autonomy and improves their quality of life.	34	468	364	416.0	66.99
Within the Hospital Network, holistic care requires recognizing subjects' needs to adapt the care environment.	3	460	369	414.5	73.81
To strengthen interaction and the caring bond with users of the Hospital Network services, health team members must engage in collaborative activities with social sensitivity.	9	454	369	411.5	63.89
The Hospital Network environment must contribute to improving the quality of life of workers and users.	32	479	313	396.0	117.23
In the Hospital Network, adequate working conditions, incentives, and the assurance of healthcare professional's well-being must be part of an environment conducive to care.	28	482	302	392.0	127.78
To ensure quality care, nursing within the Hospital Network must have adequate human and technological resources, along with clear processes and well-defined standards for managing the conditions of the subjects of care.	2	469	300	384.5	125.29
The Hospital Network needs adequate supplies to facilitate healthcare professionals’ work, achieve the goals of care, and contribute to the quality of life of its users.	31	379	302	340.5	119.71

* IMP: Level of importance for nursing; ** GV: Level of nursing governance. ***SD: Standard Deviation.

The internal validation was conducted through a presentation of the model by the research group to 691 nursing staff members within the Hospital Network. This group represented 82.54% of the total staff, including professionals and technicians. Overall, the model was positively received, and the staff expressed a sense of identification with it. 

The external validation was conducted by the University's Nursing Care Research Group, which analyzed the nursing model’s content and confirmed that it aligned with international standards for this type of theoretical development. 

Once validated, the model was presented to the Hospital Network's executive managers, who recognized its alignment with the Hospital Network’s mission and development plan. 

## Discussion

The Leadership in Compassionate and Safe Care nursing model was developed through an academic-service partnership, considering the humanistic view of the Hospital Network. It sought to guide nurses' work in research, practice, and education. This development from a partnership has proven to be effective^20^. 

Professional nursing practice models are valuable guides for nursing practice qualification^21^. These models can guide educational activities within a teaching hospital^22^, particularly during periods of organizational change^23^. However, there are no known models for guiding nursing practice across a hospital network that encompasses different levels of care, as the one proposed in this study. 

The model "Leadership in Compassionate and Safe Care" emerged from a participatory exercise involving nurses from the Hospital Network. As its name suggests, it integrates leadership, compassion, and safe care. This integration is evident in previous studies conducted from different perspectives. 

 A review of the literature on nursing leadership practice points out that, while emotional intelligence is valuable in this field, helping nursing to fulfill the purpose of their practice, the focus of leadership should be on the person receiving care rather than the professional^24^. This study acknowledges that both people receiving care and nurses themselves are subjects of care and, therefore, seeks to focus nursing leadership on the person being cared for while also not forgetting the professional’s own development. 

The exploration of leadership development from professional training indicates that it includes abilities such as explaining, fostering the necessary unity in work, motivating, representing the group externally, serving as a symbol, affirming values, renewing, anticipating goals, and managing. According to the authors, this development is based on the dimensions of knowing, doing, and being within the context. In this regard, promoting nursing leadership through implementing a model allows the internalization of this capacity and the practical application of skills. It also helps the training of new talent, enhances the selection of strategies, and advances research, ultimately enabling a better response to contextual demands^25^. 

When leadership is shared, one of the main conditions for nursing autonomy is given, that is, the ability to use their own competence and make the best possible decisions in practice, exercising intra- and interprofessional collaboration within a healthy work environment^26^. According to these considerations, this study, as evidenced by the collective participation of nurses in each phase, involved the nursing staff of the Hospital Network in developing the guidelines for the model’s construction so that their perceptions could be considered and their participation encouraged. 

Studies focused on compassionate care report similar findings. Compassionate nursing care has been conceptualized as the sum of empathy and efforts to alleviate individuals’ suffering while respecting their uniqueness and applying appropriate communication and therapeutics. According to the authors, compassionate care requires a positive practice environment with adequate resources to meet its demands. This type of care is reciprocal, benefiting all parties involved; that is, it is associated with nursing leadership in service and is basic not only for subjects of care but also for nurses and professionals in training^27^. 

Subsequent studies complement this definition by arguing that compassion should be considered at four levels: the self, manager, team, and organization. Evidence suggests that compassionate care positively affects clinical leadership and confidence in leading change in practice. Compassionate care helps maintain the patient's privacy, strengthens empathy, and acknowledges uniqueness. Additionally, it contributes to greater job satisfaction, improved sense of well-being, and greater pride in the nursing profession^28^. 

Compassionate care in healthcare systems encompasses ethical, professional, communicative, humanistic, and spiritual dimensions, along with its positive effects on subjects of care. These authors point out that there are certain factors either promoting or hindering the practice of compassionate care that should be taken into account in each setting, including the nurse's personal characteristics, patient behavior, and environmental aspects such as workload, institutional culture, and the value placed on compassionate care. For these authors, compassionate care should bridge the gap between theory and practice; therefore, it is important to work in academic-service partnerships and measure the long-term impact of these initiatives on nursing leadership, patient outcomes, nurses, and the healthcare organization^29^. 

To implement compassionate care, ensuring support, regulations, adequate resources, and the participation of nursing managers is necessary^30^. Additionally, the responsibility for providing compassionate care should not solely be on individual nurses. Care must be facilitated by the right circumstances to be able to offer and engage in compassionate interactions with patients and their families. Therefore, organizations—not just individuals—need to take responsibility for sustaining compassionate care delivery^31^. 

The conceptualization and study of safe patient care and the skills required to ensure it points out that these include patient-centered care and assertive communication. The authors report that a nurse's capacity for emotional intelligence—required for leadership—and the patient's perception of compassionate care can positively impact safe patient care^32^. 

Further important is the identification of contextual factors affecting compassionate care, compassionate care actions, and compassionate care consequences as part of a guiding model for nursing practice in this field^33^, as is evident in the development of this study. Identifying contextual factors is considered essential for strengthening nursing training and advancing nursing knowledge development^34^,^35^. This coincides with the intention of the teaching Hospital Network where this research was conducted. 

This study aligns with various authors’ approaches that integrate compassionate care, nursing leadership, and the contextual safety conditions necessary for quality nursing care^36^. The model developed by the research group views compassionate care as an opportunity for nurses to strengthen the caring bond through a cordial approach, an inclusive attitude, and supportive behavior that strengthen the autonomy of those they care for within the context of the Hospital Network. 

In summary, the proposed model for the Hospital Network, "Leadership in Compassionate and Safe Care," brings together parameters suggested by nursing literature on compassionate care, safety, and leadership in a single theoretical outline to guide practice, training, and research. 

The scope in which the developed model can be applied is considered a limitation of this study. However, the results could be useful to guide the construction of other models in hospital networks of a similar nature. 

## Conclusions

The nursing professional practice model, "Leadership in Compassionate and Safe Care," developed in this study, has made it possible to make visible, communicate, and guide nursing practice within the Hospital Network in response to its humanistic philosophy. This model meets internal consistency and acceptance criteria, has been peer-approved, and meets international standards for theoretical constructions of this kind. Its creation—based on an academic-service partnership, guided by the University's recommendations for such developments, and shaped by the collective participation of the members of the Hospital Network— facilitated its acceptance. It also helped bridge the gap between theory and practice and strengthened nursing research. In this way, the model constitutes a valuable guide for qualifying nursing knowledge and practice. It will be a challenge to continue with this construction by identifying the indicators required to make visible, evaluate, and continuously improve nursing care with leadership, compassion, and safety for the Hospital Network’s users. 
